# Supplementary material for: Effects of Resource Availability and Antibiotic Residues on Intestinal Antibiotic Resistance in Bellamya aeruginosa
Source: Microorganisms. 2023 Mar 16;11(3):765. doi: 10.3390/microorganisms11030765 (PMC10058807; doi:10.3390/microorganisms11030765)
Supplement: Supplementary file 1 [file microorganisms-11-00765-s001.zip › microorganisms-2263048-supplementary.pdf]

Table S1. Concentrations of florfenicol in water (μg/L).

Treatment	0 days (after water replacement)	84 days (before water replacement)
HN	7.28 ± 5.05	3213.05 ± 616.03
HA	7.28 ± 5.05	4304.78 ± 345.13
LN	10.47 ± 6.14	124.033 ± 31.46
LA	10.47 ± 6.14	4550.68 ± 471.28

Table S2. Formulation and proximate composition of commercial feed

Ingredient (g/100 g diet)	Basal diet
Protein	65
Fat	6
Ash	6
Water	6
Fibre	4
Calcium	0.9
Phosphorus	0.5
Probiotics	10000 CFU/g
Vitamin A	10000 IU/g
Vitamin D3	2000 IU/g
Vitamin C	600 IU/g
Vitamin E	100 IU/g
Vitamin B1	30 IU/g
Vitamin B12	5 IU/g

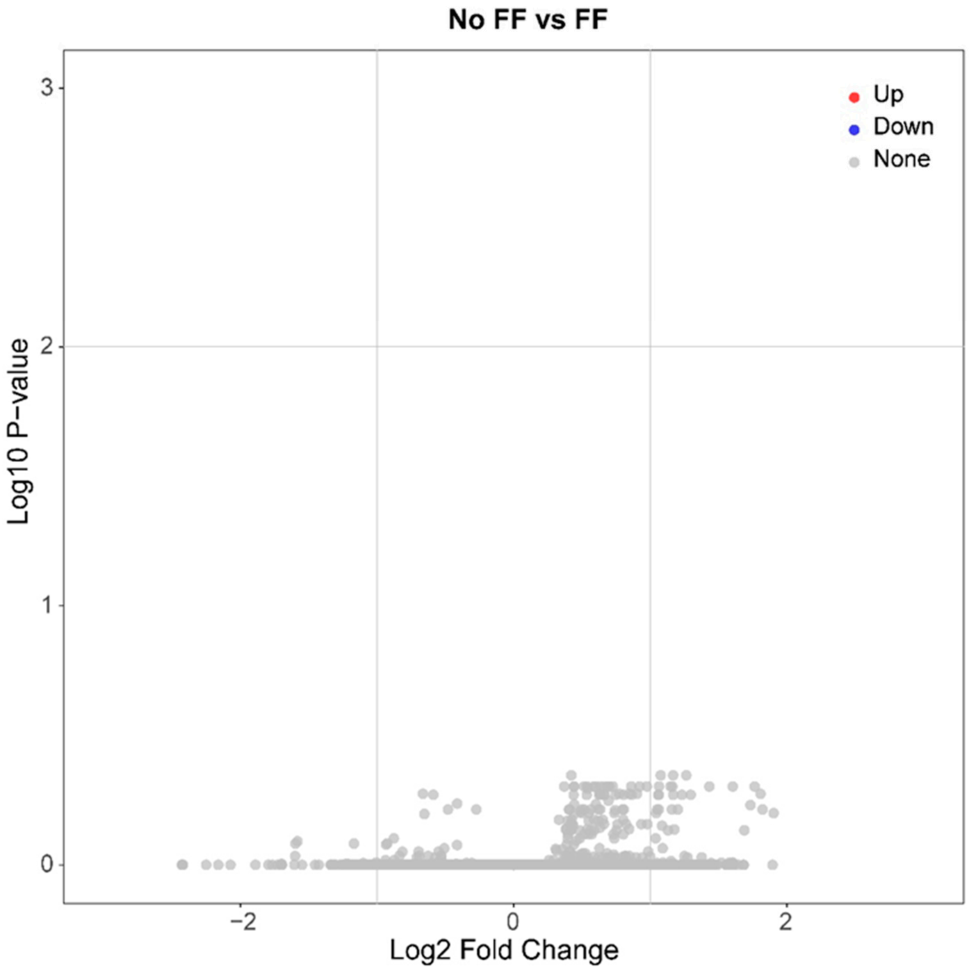

Figure S1. Impacts of florfenical on functional pathways of *Bellamya aeruginosa* intestinal bacteria.
